# Endocortical Trabecularization in Acromegaly: The Cause for the Paradoxically Increased Vertebral Fracture Risk?

**DOI:** 10.1002/jbm4.10787

**Published:** 2023-08-11

**Authors:** Ansgar Heck, Kristin Godang, Tove Lekva, Kjersti Norman Markussen, Sara De Vincentis, Thor Ueland, Jens Bollerslev

**Affiliations:** ^1^ Section of Specialized Endocrinology, Medical Clinic Oslo University Hospital Oslo Norway; ^2^ Research Institute of Internal Medicine Oslo University Hospital Oslo Norway; ^3^ Faculty of Medicine University of Oslo Oslo Norway; ^4^ Unit of Endocrinology, Department of Biomedical, Metabolic and Neural Sciences University of Modena and Reggio Emilia Modena Italy

**Keywords:** DXA, hip structural analysis, remodeling, TBS, trabecular separation

## Abstract

Growth hormone (GH) is nonphysiologically increased in acromegaly, stimulating target tissues directly and indirectly via insulin‐like growth factor type 1 (IGF‐1). Despite GH having anabolic effects on bone growth and renewal, the risk of vertebral fractures is paradoxically increased in acromegaly. We hypothesized that bone tissue compartments were differentially affected by hormonal alterations in active and controlled acromegaly. We aimed to study the effect of sex and gonadal status on long‐term outcome of bone mass and structure to understand the biomechanical competence of bone. We followed 62 patients with newly diagnosed acromegaly longitudinally (median 4.8 years after pituitary surgery) to investigate changes assessed by dual X‐ray absorptiometry (DXA), trabecular bone score (TBS), and hip structure analysis (HSA). At diagnosis, patients had increased bone mineral density (BMD) in most compartments compared with normative data (Z‐scores). Conversely, TBS Z‐score was decreased (Z = −0.64 (SD 1.73), *p* = 0.028). Following treatment of acromegaly, BMD increased further in compartments containing predominantly trabecular bone, such as the lumbar spine, in eugonadal and male subjects, while compartments with predominantly cortical bone, such as the hip and femoral neck, were unchanged. Total body measurements showed further increase in BMD independent of sex and gonadal status. TBS did not change. HSA revealed a significant decrease in cortical thickness in both sexes independent of gonadal status, whereas the overall size of bone (hip axis length and neck width) did not change over time. In conclusion, patients with acromegaly had increased bone mass and dimensions by DXA. Following normalization of disease activity, BMD increased mainly in compartments rich in trabecular bone, reflecting a closure of the remodeling space. However, HSA revealed a significant decrease in cortical thickness, implying endocortical trabecularization, potentially explaining the increased risk for incident vertebral fractures following treatment. © 2023 The Authors. *JBMR Plus* published by Wiley Periodicals LLC on behalf of American Society for Bone and Mineral Research.

## Graphical abstract

Normalization of disease activity was followed by an increase in BMD in lumbar spine (L1–L4) without changes in TBS, but a marked decrease in CBT in Femoral Neck and Calcar Region. These data imply endocortical trabecularization that could be linked to the high incidence of vertebral fractures following treatment of acromegaly.

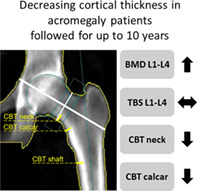

## Introduction

Bone dimensions are increased in active acromegaly, primarily due to enhanced periosteal apposition, theoretically leading to a decreased fracture risk.^(^ ^1^, ^2^ ^)^ Growth hormone (GH) exerts direct effects through interaction with the GH receptor in target tissues and indirectly mediated via insulin‐like growth factor 1 (IGF‐1) produced by the regulation of GH, mainly in the liver (the somatomedin hypothesis),^(^ ^3^ ^)^ but also in peripheral target tissues including bone.^(^ ^4^, ^5^ ^)^ In active acromegaly, nonphysiological levels of GH and IGF‐1 have a major impact on bone homeostasis. Indeed, bone turnover is increased, where bone resorption and formation are coupled, as in normal physiology,^(^ ^5^, ^6^ ^)^ thereby causing an enlargement of the bone remodeling space and trabecular separation.^(^ ^7^, ^8^ ^)^ As a result, both bone quality and quantity are compromised in patients with active acromegaly.

In an early ex vivo study on biomechanical competence of iliac trabecular bone in active acromegaly, trabecular bone strength decreased compared to normal controls, even after adjusting for the reduced trabecular bone mass in the patient population.^(^ ^9^ ^)^ In accordance with this finding, Mazziotti and coworkers demonstrated an elevated prevalence of vertebral fractures in both postmenopausal women and men with acromegaly.^(^ ^10^, ^11^, ^12^ ^)^ Moreover, meta‐analysis by the same group reported that the risk of vertebral fractures was higher in men compared to women, in hypogonadal compared to eugonadal patients, and in patients with active disease compared to control patients.^(^ ^13^ ^)^ Another recent meta‐analysis confirmed that the risk for vertebral fractures was sevenfold elevated in patients with acromegaly compared to controls.^(^ ^14^ ^)^ Taken together, patients with acromegaly possess an increased fracture risk, especially in trabecular bone, despite an increase in overall bone size and potentially enlarged cortical thickness, and thereby an improved moment of inertia.^(^ ^15^ ^)^

The effects of active acromegaly on the different compartments and envelopes of bone at the organ level are scarcely studied. Recent experimental studies indicated sexual dimorphism on the skeletal effects of chronic GH and IGF‐1 excess^(^ ^16^ ^)^ complicated by local GH‐stimulated IGF‐1 and corresponding IGF‐binding protein production.^(^ ^5^, ^17^ ^)^ Based on bone mineral density (BMD) measurements of the hip (TH) and femoral neck (FN) by dual X‐ray absorptiometry (DXA), structural information can be derived (hip structure analysis [HSA])^(^ ^18^, ^19^ ^)^ and be of importance for understanding the biomechanical competence of the appendicular skeleton and the interplay between the different envelopes in acromegaly.^(^ ^20^ ^)^

Whereas BMD by DXA gives a quantitative assessment of the investigated region (ex the lumbar spine [LS]), trabecular bone score (TBS) provides an estimation of bone microarchitecture, potentially reflecting the biomechanical properties of trabecular bone.^(^ ^21^, ^22^ ^)^ As reviewed recently, studies of TBS in acromegaly have shown inconsistent results.^(^ ^14^ ^)^ In a longitudinal, 1‐year study of 48 patients, we found that successful treatment was followed by an expected decrease in biochemical markers of bone turnover and thereby an increase in BMD in LS, but a paradoxical decrease in TBS, most pronounced in men and eugonadal patients.^(^ ^23^ ^)^ The unexpected nature of these findings, potentially partly related to technical issues such as changes in abdominal body composition by treatment of the underlying disease, prompted us to further evaluate TBS in larger and longer‐lasting studies of acromegaly.^(^ ^23^ ^)^

Here we hypothesized that bone tissue and compartments were differentially affected by the various hormonal alterations in active and controlled acromegaly, which is important for long‐term skeletal health. Thus, we aimed to investigate the longitudinal changes of bone tissue assessed by DXA (BMD), has, and TBS at baseline and following individualized and standardized management for 3 years after surgery for acromegaly and beyond. Furthermore, the secondary aims of the study were to examine the effects of sex and gonadal status on the long‐term outcome of indices of bone mass and structure.

## Material and Methods

### Patient characteristics and management

During the period from 2006 to 2016, 62 patients with active acromegaly were diagnosed and worked up by a standard protocol and enrolled in the present study. Inclusion criteria were newly diagnosed acromegaly, available DXA scan at baseline, and long‐term follow‐up visit (3–8 years after pituitary surgery). Diagnosis was based on clinical symptoms, elevated age‐adjusted IGF‐1 levels, and a lack of sufficient suppression of GH during a standard oral glucose tolerance test (OGTT), as described previously.^(^ ^23^ ^)^

Oslo University Hospital (OUS), Rikshospitalet, is a tertiary care center covering specialized endocrine and neurosurgical services for about three million inhabitants. During the inclusion period, 133 new patients were assessed in the Section of Specialized Endocrinology, and baseline DXA scans were available for 97 of these. After exclusion of patients with other diseases with potential impact on bone metabolism (ex primary hyperparathyroidism) or unavailable scans at the long‐term follow‐up, these 62 patients were included in the analyses. When several scans were available during follow‐up, the scan closest to the 5‐year postoperative date was included.

Hypogonadism was defined as described.^(^ ^23^ ^)^ Briefly, patients were defined as hypogonadal if they did not receive treatment with sex steroids and had plasma estradiol levels (female) or testosterone and/or free testosterone index (total testosterone/SHBG × 10; male) below the reference values. Patients changing their gonadal status during the study period were classified according to longest time interval during the study.

### Biochemistry

We measured plasma GH by immunoassay (IMMULITE 2000; Siemens Healthcare GmbH, Erlangen, Germany) until 2015, then Roche Modular E170 for the years 2015–2016. Later, we used a Roche Cobas® e602 (Roche Diagnostics, Basel, Switzerland).^(^ ^24^ ^)^ Sex steroids, sex hormone binding globulin (SHBG), luteinizing hormone (LH), and follicle‐stimulating hormone (FSH) were assayed in plasma by Roche Modular E170 until 2016 and thereafter by the same platform as described earlier (Roche Cobas® e602).

Serum IGF‐1 was analyzed by immunoassay (IMMULITE 2000; Siemens Healthcare GmbH, Erlangen, Germany)^(^ ^24^ ^)^ until November 2016, then a noncompetitive immuno luminometric method was established (Immulite 2000xpi from Siemens Healthineers). Over the years, different methods were used; however, at every change of method, cross‐calibration was done and, if necessary, factorial adjustments performed.

As part of the daily routine, the samples were measured consecutively, by accredited methods, at the Department of Medical Biochemistry, OUS, using standard analysis protocols.

### DXA measurements

BMD was determined using DXA. A narrow fan beam (GE Healthcare Lunar Prodigy, Madison, WI, USA) densitometer was used and all scans analyzed using software version 16 [SP2]. We analyzed anterior–posterior (AP) lumbar spine (LS; L_1_–L_4_), bilateral proximal femur, dual total hip (TH), and dual femoral neck (FN) and present BMD for all these regions.^(^ ^23^ ^)^ Absolute BMD values (g/cm^2^) and Z‐scores were estimated by comparison to the reference population incorporated in the software and previously shown to be suitable for clinical use in Norway.^(^ ^25^ ^)^ We calibrated the densitometer daily,^(^ ^26^ ^)^ where the short‐ and long‐term coefficients of variation for our hardware were 0.8% and 1.4%, respectively.^(^ ^27^ ^)^

### Assessment of trabecular bone score

As described previously, lumbar spine TBS parameters were extracted from DXA L_1_–L_4_ images using TBS iNsight software (version 3.0.3.0; Medimaps Group, Geneva, Switzerland).^(^ ^23^ ^)^ KG, an International Society for Clinical Densiometry (ISCD)‐certified densitometry technologist, performed all the analyses based on the original scans, as described.^(^ ^23^ ^)^ Compressed vertebrae were excluded from the analyses. TBSs for age and gender (TBS Z‐score) acquired from the manufacturer's (TBS iNsight) reference database (only available for females ≥45 years and males ≥40 years of age) were estimated.

### Hip structure analysis

HSAs were performed as described previously.^(^ ^20^, ^28^ ^)^ As originally defined by Martin and Burr,^(^ ^29^ ^)^ the HSA program assesses the structural geometry of the proximal femur from bone mineral mass and dimensional data based on the DXA‐derived images of the hip. Lines of pixel value pass over the bone axis in the bone mass image, and the HSA program presents fully mineralized adult cortical bone. Cortical bone thickness (CBT) was measured in three regions of the proximal femur: (1) narrow neck (CBT neck), (2) the intertrochanteric calcar (CBT calcar), and (3) the femoral shaft (CBT shaft). Further, the program analyzed the femur neck width (NW) at the narrowest point (NW) and hip axis length (HAL). HAL is defined as the distance from the greater trochanter to the inner pelvic rim.^(^ ^30^ ^)^ The program automatically defined all measurement sites. We deleted obvious errors in the program (extremely high or low values), including obvious measurement errors (e.g., due to positioning errors, lack of inward twisting of ankle, incomplete scans, and other artifacts).^(^ ^29^ ^)^

### Statistics

Statistical analyses were conducted using SPSS for Windows, version 21.0 (SPSS, Chicago, IL, USA). In general, data were expressed as mean ± SD when normally distributed and median (interquartile range [IQR]: 25th–75th percentile) when skewed. Comparison between the time points of acromegaly was performed using a *t* test or Mann–Whitney U depending on the distribution and a chi‐squared test for categorical variables. Differences in the temporal course of bone indices from baseline to 1‐ and 5‐year postoperative (PO) control according to sex and gonadal status were evaluated by mixed model analysis focusing on time and group effects (i.e., sex or gonadal status) or their interaction (e.g., sex × time), adjusted for age. Data from these analyses were expressed as estimated marginal means and 95% confidence intervals (CIs). Two‐tailed *p* values <0.05 were considered significant.

### Ethics

The study was approved by the Regional Ethics Committee of the Health Region South‐East, Norway (REK nos. 15240 and 249429). All patients gave informed consent for participating in the study according to the Helsinki II Declaration.

## Results

### Patients and osteodensitometry data at baseline

As given in Table 1, the baseline demographics of the patient population show an almost equal sex distribution (54.8% men) with a mean age of 46.4 years (SD 13.9). Among the women, 50% were defined as hypogonadal (postmenopausal women included). Of the male subjects, two of 34 (6%) were defined as hypogonadal. Disease activity was confirmed by high GH levels and a mean IGF‐1 level of 2.8 (IQR: 1.8–3.6) times the upper limit of normal (ULN).

**Table 1:** Measurements at Baseline, 1‐Year and 5‐Year (Range 3–8) Postoperative Follow‐Up of 62 Patients with Acromegaly

Variables	Baseline	1‐yearPO	5‐year PO
Men/women (*n*)	34/28	29/25	34/28
Hypogonadal: m:2/w:14			
Patients on medical treatment^a^ for acromegaly (men/women) (*n*)^a^	0/0	15/10	16/7
Age at baseline (years)	46.4 (13.9)		
Weight (kg)	88.2 (19.3)	87.8 (18.6)	90.0 (17.8)
Height (m)	1.76 (0.10)	1.76 (0.10)	1.76 (0.11)
BMI (kg/m^2^)	28.1 (4.6)	28.3 (4.9)	29.0 (4.7)
*Disease activity*			
GH (μg/ml)	8.8 (4.6, 22.8)	1.1 (0.5, 2.9)^c^	0.9 (0.5, 3.0)^c^
IGF‐1 (nmol/L)	113 (83, 139)	30 (23, 49)c	21 (16, 29)^c^ ^,^ ^d^
IGF‐1/ULN	2.8 (1.8, 3.6)	0.9 (0.7, 1.3)^c^	0.9 (0.7, 1.1)^c^
*DXA*			
Lumbar spine L1‐L4 BMD Z‐score	0.38 (1.71)	0.68 (1.64)^b^ ^,^ ^c^	0.86 (1.82)^b^ ^,^ ^c^ ^,^ ^d^
Dual femoral neck BMD Z‐score	0.30 (1.06)^b^	0.40 (1.10)^b^ ^,^ ^c^	0.37 (1.12)^b^
Dual total hip BMD Z‐score	0.38 (1.13)^b^	0.53 (1.10)^b^ ^,^ ^c^	0.44 (1.21)^b^
Total BMD Z‐score	0.66 (1.38)^b^	1.16 (1.53)^b^ ^,^ ^c^	1.30 (1.52)^b^ ^,^ ^c^ ^,^ ^d^

^a^ Medical treatment including somatostatin analogues, Dopamine agonists, GH antagonists.

^b^ *p* < 0.05 Z score in patients with acromegaly compared to zero (=mean value of reference population for Z‐score).

^c^ *p* < 0.05 change between baseline to 1‐year PO and baseline to 5‐year PO.

^d^ *p* < 0.05 change between 1‐year to 5‐year PO.

Compared to the reference population (Z‐scores, Table 1), BMD in the LS was unaltered at baseline, but higher in both hip compartments (FN and TH, *p* < 0.05) and in total bone (TB, *p* < 0.05). TBS Z‐scores were only available for 38 patients due to limitations in the provided software. Mean TBS Z‐score for this group was lower than controls −0.64 (SD 1.73), *p* = 0.028.

### Patients during follow‐up

Median long‐term follow‐up time after pituitary surgery was 4.8 years (IQR: 3.8–5.6; range 3.0–8.0). A modest increase in weight and BMI during this period was observed, whereas median GH and IGF‐1 levels fell significantly to normal levels (*p* < 0.05 for both, Table 1).

### Osteodensitometry during follow‐up

**Fig. 1:**
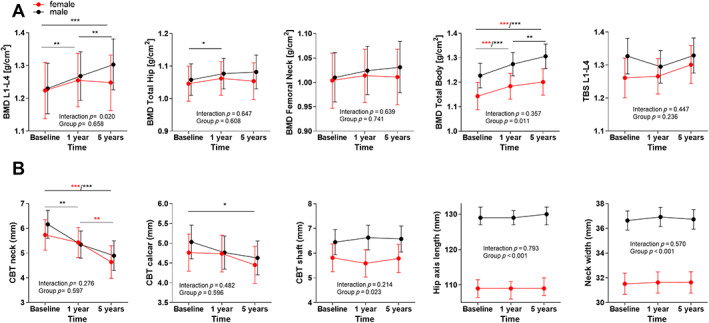
Indices of bone mass and structure before any treatment (Baseline), 1 year postoperatively (1y) and after long term follow up (5year: 4.8 years; IQR: 3.8–5.6) in female patients (red) vs. male patients (black) (Mixed model analyses). *p < 0.05, **p < 0.01, ***p < 0.001.

**Fig. 2:**
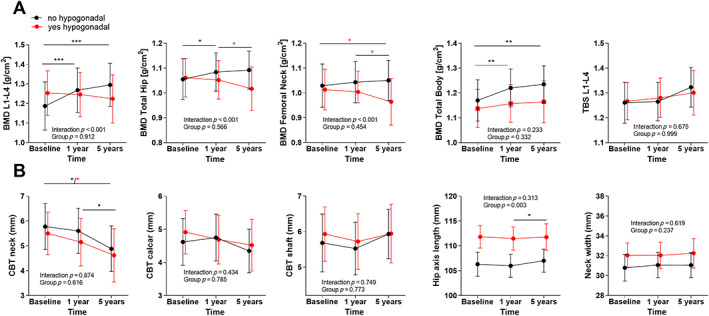
Indices of bone mass and structure in women stratified by gonadal status: Before any treatment (Baseline), 1 year postoperatively (1y) and after long term follow up (5years: 4.8 years; IQR: 3.8–5.6) in hypogonadal women (red) vs. non‐hypogonadal women (black). (Mixed model analyses).*p < 0.05, **p < 0.01, ***p < 0.001.

Following treatment, LS BMD (g/cm^2^) increased in men at 1 year (*p* < 0.01), with a further increase at long‐term follow‐up (*p* < 0.01), but not in women (Fig. 1*A*). A similar temporal increase in BMD LS was observed in eugonadal women postoperatively, but not in hypogoadal subjects (Fig. 2*A*), with an overall difference between the groups (mixed model regression, *p* < 0.001).

For the femoral sites (TH and FN), BMD increased for TH only in male and eugonadal female patients (TH) (Fig. 1*A* and 2*A*; *p* < 0.05). There was no interaction according to sex, but we observed a decline in BMD in both femoral sites in hypogonadal (*p* < 0.001), but not in eugonadal women (Fig. 2*A*).

BMD TB was higher in men compared to women at baseline and throughout follow‐up (Fig 1*A*, *p* = 0.011) and increased significantly in both groups without temporal differences between sexes. BMD TB women increased in eugonadal but not hypogonadal women (Fig 2*A*).

TBS did not change significantly over time and did not differ if stratified by sex or gonadal status (Fig. 1 *A* and Fig. 2*A*).

### Hip structural analyses

The HSA analyses are shown in Figures 1*B* and 2*B*, stratified by sex (Fig. 1*B*) and gonadal status in women (Fig. 2*B*). As expected, male patients had higher measures in CBT in the shaft, for HAL, and NW. CBT in the neck and in the calcar region did not differ between sexes but decreased significantly with time in both sexes, for calcar only in men (*p* < 0.05, see Fig. 1*B*). CBT development did not differ between eugonadal and hypogonadal women (Fig. 2*B*). Surprisingly, HAL was longer in hypogonadal than eugonadal women. Additional analyses on height in eu‐ versus hypogonadal women did not show significant changes (data not shown).

## Discussion

In this observational study of a well‐characterized cohort of newly diagnosed patients with acromegaly and long‐term follow‐up examinations, we demonstrated that patients with active disease had increased bone mass by DXA compared with normative data in several compartments, primarily reflecting cortical bone‐rich areas. At a median of 5 years after pituitary surgery, bone mass increased further in the LS compartment in men and eugonadal women, and in TB for both sexes. Whereas TBS did not change with time, cortical thickness decreased significantly in both sexes, independently of gonadal status. Men had markedly larger hip dimensions compared to women.

Vertebral fractures are frequently silent and demonstrated by chance or during systematic studies. As outlined, the prevalence of incident fractures increases in patients with acromegaly or with a history of previous active acromegaly,^(^ ^7^, ^10^, ^11^, ^12^, ^14^, ^31^, ^32^, ^33^ ^)^ despite an increase in the bone area of vertebral bodies. The overall bone fragility is potentially related to microstructure alterations in trabecular thickness, trabecular separation, and cortical bone porosity.^(^ ^1^, ^5^, ^9^, ^34^, ^35^ ^)^ Moreover, bone turnover, but not BMD, is associated with biomarkers of disease activity.^(^ ^5^, ^23^ ^)^ Our present data support the previous findings, with an increase in bone mass in most compartments in active disease and a further increase following normalization of GH metabolism. The normalization of bone turnover reduces the bone remodeling space, thereby increasing BMD^(^ ^23^, ^36^, ^37^ ^)^ and potentially improving fracture risk.^(^ ^14^ ^)^

TBS is a DXA‐derived estimate of the microstructure of the trabeculae in vertebral bodies and, thus, complementary to BMD.^(^ ^21^, ^22^ ^)^ At baseline, TBS was reduced compared to normative data, but should be interpreted with caution as data were only available for about two thirds of the population due to software limitations. In contrast to our previous study,^(^ ^23^ ^)^ here, based on a larger cohort followed for a longer period, we found that TBS did not change significantly over time. Moreover, we could not demonstrate a sex or gonadal status difference in TBS. TBS in acromegaly has given conflicting results and seems to be independent of bone mass by DXA (BMD).^(^ ^14^, ^23^, ^38^ ^)^ The reason might be multifactorial and related technical reasons due to body composition and changes herein with time,^(^ ^23^ ^)^ but other confounders, such as disease activity and duration, sex steroids, and the definition of gonadal status, should be considered.^(^ ^14^, ^23^ ^)^ The bottom line is, however, the decreased biomechanical competence of pure trabecular bone, as we showed previously ex vivo in patients with active disease, demonstrating the reduced pure trabecular bone mass of biopsies by peripheral quantitative computed tomography (pQCT).^(^ ^9^ ^)^ Even when normalized for bone mass, the strength of the bone core was still reduced.^(^ ^9^ ^)^ These findings were subsequently supported by clinical observations of increased vertebral fracture risk in both sexes by the Brescia group.^(^ ^10^, ^11^ ^)^ The discrepancy in our previously shown changes of BMD and TBS shortly following treatment^(^ ^23^ ^)^ and here only as a potential weak and insignificant trend might be related to the decrease in bone turnover by treatment. Bone turnover is increased and synchronized (coupled) in active disease,^(^ ^5^, ^7^, ^8^ ^)^ and GH is in principle anabolic to bone, as reflected by the periosteal apposition leading to the enlarged bone area.^(^ ^5^ ^)^ The change in turnover following treatment and normalization of disease activity may divergently influence the remodeling cycle and various bone envelopes, having different effects on bone mass (BMD) and microstructure (TBS)^(^ ^7^ ^)^ (see following discussion).

As expected, the total dimensions of the hip based on HSA were larger in men compared to women and did not change over time, even in this larger cohort with prolonged observation following normalization of disease activity.^(^ ^20^ ^)^ Though we have no normative data for HSA, our previous study demonstrated that these measures increased compared with patients with nonfunctioning pituitary adenomas (NFPAs).^(^ ^20^ ^)^ The data were in agreement with previous studies of bone dimensions in acromegaly^(^ ^5^ ^)^ and the discussion of the increased moment of inertia,^(^ ^15^, ^29^ ^)^ potentially being protective for appendicular fractures in these patients.^(^ ^2^ ^)^ Moreover, cortical thickness by HSA was increased in acromegaly compared with the NFPA patients and changed differently by group during 1 year of follow‐up in the hip region.^(^ ^20^ ^)^ These data were further developed in the present study. The CBT analyses were in general not dependent on sex or gonadal status in the hip region (CBT neck and CBT calcar) but lower in females in the shaft region, reflecting the women's smaller body dimensions. While we noted a large difference in HAL and NW in relation to sex, gonadal status surprisingly showed slightly higher HAL in hypogonadal women. Previously, Nissen et al. reported that HAL increased with age in women, but not in men.^(^ ^39^ ^)^ Since the mixed model calculations in the present study were age adjusted, we did not observe any longitudinal change in HAL, and in all likelihood there was no effect of the normalization of GH activity. We therefore hypothesize that hypogonadism is associated with larger HAL rather than by age itself.

With normalization of disease activity and prolonged observation, CBT in the femoral neck decreased significantly, independent of sex and gonadal status. Because we found no change in NW by treatment and time, this can be explained by an enlargement of the trabecular space, so‐called endocortical trabecularization, as seen in primary hyperparathyroidism^(^ ^40^ ^)^ and postmenopausal osteoporosis.^(^ ^41^ ^)^ This process will have biomechanical consequences, both for peripheral fractures and, most importantly, trabecular bone in vertebral bodies due to trabecular separation. The process may also be linked to the paradoxical changes in TBS with management and time shown in our previous study^(^ ^20^ ^)^ and here compared with the development of BMD.

The strength of our study is the observational design of a well‐characterized cohort of newly diagnosed patients with acromegaly worked up by a standard protocol.^(^ ^42^ ^)^ Further, the patients were followed within the protocol for an extended period. A limitations is the lack of a control group, as we rather refer to normative data (Z‐scores) validated for Norwegian patients.^(^ ^25^ ^)^ In addition, there were only two hypogonadal men, making it difficult to fully evaluate the effect of hypogonadism on the temporal trajectory of bone mass and dimensions in hypogonadal men. Six patients received glucocorticoid replacement therapy in physiological doses during the study period. Only a very small number of patients received bone active drugs, and we did not adjust for that. Not all patients with a baseline scan had follow‐up exams, and regions of interest that could not be analyzed quantitatively were excluded. We cannot exclude the possibility that this could have affected the results. The lack of hard endpoints (fractures) is another limitation.

In conclusion, patients with acromegaly had increased bone mass and dimensions, as determined by DXA. Following normalization of disease activity, BMD increased, further reflecting a closure of the bone remodeling space. This change seems not to have reached a new steady state in the 1‐ to 5‐year period following treatment. The increased bone dimensions in acromegaly are of importance for peripheral bone strength. However, HSA revealed a significant decrease in cortical thickness, implying endocortical trabecularization. This process might explain the paradoxical findings of TBS in acromegaly and the increased risk for incident vertebral fractures following treatment.

## Author Contributions

**Ansgar Heck:** Conceptualization; data curation; formal analysis; methodology; project administration; supervision; writing – original draft; writing – review and editing. **Kristin Godang:** Conceptualization; data curation; investigation; methodology; project administration; supervision; writing – review and editing. **Tove Lekva:** Data curation; formal analysis; methodology; visualization; writing – review and editing. **Kjersti Norman Markussen:** Investigation; writing – review and editing. **Sara De Vincentis:** Visualization; writing – original draft; writing – review and editing. **Thor Ueland:** Formal analysis; methodology; writing – review and editing. **Jens Bollerslev:** Conceptualization; methodology; supervision; writing – original draft; writing – review and editing.

## Conflict of Interest Statement

All the authors declare no potential conflicts of interest.

### Peer Review

The peer review history for this article is available at https://www.webofscience.com/api/gateway/wos/peer-review/10.1002/jbm4.10787.

## Data Availability

Due to privacy regulations, data are not publicly available.
